# Mental health of persons with disabilities during the COVID-19 pandemic in Bangladesh

**DOI:** 10.1371/journal.pone.0322218

**Published:** 2025-04-29

**Authors:** Md. Omar Faruk, Md Abu Bakkar Siddik, Kamal Uddin Ahmed Chowdhury, Nazmul Bari, Shafayet Hossain, Sumaiya Noor, Mohammad Rezaul Alam, Taslima Akter, Ben Adams, Manivone Thikeo, Mohammad Meshbahur Rahman

**Affiliations:** 1 Centre for Disability in Development, Savar, Dhaka, Bangladesh; 2 Department of Clinical Psychology, University of Dhaka, Dhaka, Bangladesh; 3 The Center for Social Policy and Justice, Dhaka, Bangladesh; 4 School of the Environment, Nanjing University, Nanjing, China; 5 Department of Development Studies, Daffodil International University, Dhaka, Bangladesh; 6 Deakin University, Geelong, Victoria, Australia; 7 CBM Global Disability Inclusion, Richmond Victoria, Australia; 8 Department of Biostatistics, National Institute of Preventive and Social Medicine (NIPSOM), Dhaka, Bangladesh; Jashore University of Science and Technology (JUST), BANGLADESH

## Abstract

**Background:**

The COVID-19 pandemic has impacted the mental health of people across the world, including those with disabilities in Bangladesh. However, very little research exists that has explored the mental health problems experienced by persons with disabilities in rural and urban areas of Bangladesh. This study aimed to investigate the prevalence and associated factors of common mental health problems in persons with disabilities in rural and urban areas of Bangladesh.

**Methods:**

A cross-sectional survey using the Bangla Depression Anxiety Stress Scale-21 (BDASS-21) with sociodemographic was conducted among 950 participants with varying types of disabilities in Dhaka, Narayanganj, and Gazipur. Descriptive and inferential statistical analyses were used to measure the effects.

**Results:**

The prevalence of moderate to extremely severe depression, anxiety, and stress among participants was 67.6%, 72.6%, and 49.5%, respectively. Urban participants exhibited significantly higher levels of depression (76.6% in Dhaka), anxiety (86.1% in Dhaka), and stress (32.1% in Dhaka) compared to their rural counterparts (depression: 86.16%, anxiety: 91.07%, stress: 97.77% in Gazipur). Gender differences were observed in anxiety, with females reporting higher anxiety levels than males (p<0.05). Age and geographical location were significantly associated with stress (p<0.042 and p<0.001, respectively), with those reporting higher anxiety also experiencing greater stress (p<0.001). Specific disabilities, such as visual disabilities, were linked to higher stress levels, while depression and anxiety did not show significant associations with demographic factors or disability type.

**Conclusion:**

Results highlight the prevalence of common mental health problems among persons with disabilities in Bangladesh. The findings can contribute to the development of appropriate public health intervention plans taking into consideration persons with disabilities, especially during emergencies.

## Introduction

The global prevalence of persons with disabilities is 15%, and 80% of adults with disabilities live in low- and middle-income countries [1]. Persons with disabilities are more vulnerable to developing mental health problems [2–6]. They also experience lower life satisfaction due to challenges in their daily lives such as access to public transportation, community mobilization, and poor healthcare systems [7]. Mental health repercussions may be higher during emergencies such as the COVID-19 pandemic. Evidence from past pandemics has shown that people with disabilities experience higher mental health problems compared to persons without disabilities [8]. Yet, studying the mental health status of persons with disabilities during the COVID-19 pandemic attracted little attention worldwide including in Bangladesh. Therefore, the present study aimed to study the common mental health problems among persons with disabilities during the pandemic in rural and urban areas of Bangladesh.

The prevalence of disability in Bangladesh is estimated to range between 1.4% and 17.5%– an estimate that excludes hearing impairments, and developmental, and learning disabilities [1]. The government of Bangladesh has enacted several provisions to support people with disabilities, but these have come under increased pressure due to lock-downs and inadequate staffing during the COVID-19 pandemic [9,10]. Bangladesh generally reports an increasing prevalence of mental health problems [11] with limited resources and trained staff to support mental health care needs. Specialized mental health care services are usually concentrated in capital cities, leaving people from rural and remote areas without mental health care. This situation is likely to be worse for those with disabilities. Insufficient statistics about the mental health status of persons with disabilities, and the lack of services in rural and urban areas, can hamper devised mental health programs during emergency circumstances, as was the case during the COVID-19 pandemic.

The restrictions after the spread of the pandemic have impacted the well-being of people across the world, including in Bangladesh, and have led to major public health concerns [12]. Evidence suggests that anxiety, depression, posttraumatic stress disorder, sleep disturbances, troubled interpersonal relationships, fear of separation from loved ones, decreased freedom, increased uncertainty, increased helplessness, and rates of suicide, child abuse, and domestic violence [13–15] were all key mental health problems and/or determinants during the pandemic [16]. These problems may be disproportionately experienced by the approximately one billion persons with disabilities around the globe who may be at risk of increased morbidity and mortality during the pandemic [17].

This study aims to examine the disproportionate impact of COVID-19 on persons with disabilities emerged from various challenges such as limited access to routine healthcare, disruptions in rehabilitation services, and the social consequences of pandemic containment measures. The enforcement of lock-downs and movement restrictions may have led to increased levels of dependency, reduced autonomy, and heightened vulnerability to physical and psychological distress. Additionally, prolonged isolation has been associated with a higher risk of abuse, including physical and sexual violence, among persons with disabilities [18,19]. Prolonged stays at home and isolation have also increased the risk of physical and sexual abuse for persons with disabilities [20]. Given the widespread impact of the pandemic on mental health, this study specifically aimed to investigate the prevalence of common mental health conditions such as anxiety, depression, and stress among persons with disabilities. Furthermore, it also sought to examine how these mental health outcomes vary across different demographic factors, including age, gender, and geographical location.

## Method

### Study design and sample size

A cross-sectional study with a purposive sampling strategy was conducted in urban areas in Dhaka (slum areas of Bauniabadh and Kafrul) and Narayanganj (North Noshingpur and Syedpur) and in rural regions of Gazipur (Chandara and Kuniyapachor) between January 15 to July 30, 2021. Urban areas where the study was conducted contained higher population density whereas rural areas had lower population density. The sample size for this study was calculated following the formula:


$$SampleSize=\frac{\frac{z^{2}.p(1-P)}{e^{2}}}{1+\frac{z^{2}.p(1-p)}{e^{2}N}}=900$$

Where,

N= 4 million; the estimated number of disabled people in the national data

Z = 1.96, Z value for 95% confidence limits,

P = Assumed prevalence (i.e., 0.50 for 50%),

e = Margin of error (e), approximately 3.27%.

A total of 1030 participants were purposively recruited for the study.

### Participants

The Persons with Disabilities Rights and Protection Act of 2013 in Bangladesh lists 12 types of disabilities [21]. However, the present study only recruited participants with physical, visual, speech, and hearing disabilities, excluding participants with cognitive impairments and deficits in visual functioning and communication. Participants with cerebral palsy and autism spectrum disorder (ASD) were excluded from the study, as they are often found to be associated with intellectual disability [22–24]. Evidence also suggests that those with ASD and intellectual disability commonly manifest deficits in visual functioning [25]. Down syndrome is also linked to marked deficits in intelligence [26]. Individuals with these conditions may have difficulties understanding and accurately responding to self-report measures, which could affect the validity and reliability of the findings. Additionally, mental health conditions in this population are often assessed using different methodologies, such as caregiver reports or clinical evaluations, rather than self-reported psychometric tools. For example, the Alzheimer’s Association stresses that to identify cognitive impairments accurately it is important to include both patient and informants [27]. Determining cognitive impairments generally require systemic approach often involving knowledgeable informants [28–30]. Given these complexities, the study focused on disabilities where self-report measures are more reliably applicable, ensuring the accuracy of the data collected.

A total of 1030 participants were purposively recruited for the study. Participants (n=80) who did not complete the entirety of the questionnaire were removed from the data analysis, which was conducted on 950 completed surveys. Participation was voluntary, and no reimbursement was provided for completing the questionnaire.

### Procedure

Data was collected by 17 trained community mental health workers. Eleven of the mental health workers had disabilities (Male-3, Female-8), while the remaining (Male-1, Female-5) did not. The data was collected through a purposive sampling method via an offline, self-report paper survey in both rural and urban areas.

The Washington Group Short Set on Functioning (WG-SS) is a widely used tool for disability data collection [31]. The 6-item tool was designed to identify people with functional limitations and has been used by various government and non-governmental organizations across the world [31]. The tool was used for disability mapping by the Organizations of Persons with Disabilities (OPD) in respective areas after the necessary translation and modification. Based on the mapping, lists of persons with disabilities were prepared which were used to recruit participants for the present study. Participants were approached by community mental health workers while making door-to-door visits. They were instructed in both forms, verbal and written. Accessible formats (e.g., braille and use of a sign language interpreter) were also used to collect the data. A signed informed consent form with accessible formats (e.g., braille for those with a visual impairment) was also obtained from the participants. The informed consent form explicitly mentioned the nature and purpose of the study, the procedures involved, and the participant’s right to withdraw. A thumb mark was used to indicate the consent of those with no literacy capabilities. Use of dignified language (e.g., “persons with disabilities” instead of “disabled persons”) was followed. Data was collected after the countrywide lockdown was lifted, and appropriate safety measures were followed during the process of data collection.

### Ethical review

The study was approved by the ethical review committee of the Department of Clinical Psychology at the University of Dhaka in Bangladesh (project ID: IR201201). The guidelines in the Declaration of Helsinki were followed during the data collection procedures. A mental health professional was available to support the participants if they encountered any difficulties during the data collection process.

## Measures

The following measures were used to conduct the study

### Sociodemographic measures

Sociodemographic measures included age, gender (male, female), type of disability (vision, hearing, multidimensional, physical, speech), and locality (Dhaka, Gazipur, Narayanganj).

### Bangla Depression Anxiety Stress Scale (BDASS-21)

The DASS-21 and its Bangla version, BDASS-21, have been extensively used to assess mental health, specifically focusing on depression, anxiety, and stress [32–37]. The BDASS-21 was selected for this study due to its established validity within the Bangladeshi population, where it has been pretested on a sample of persons with disabilities (Alim et al., 2014) and has demonstrated psychometric robustness in other studies involving adults and general populations [38].

The BDASS-21 consists of 21 items, divided into three sub-scales—depression, anxiety, and stress—each with seven items. It uses a four-point Likert scale to rate the frequency of symptoms, with response options ranging from “0” (never) to “3” (always). The total score is calculated by summing the individual responses for each subscale, and the scores are classified as follows: for depression, scores from 7 to 10 are moderate, 11–13 are severe, and 14+ are extremely severe; for anxiety, scores of 6–7 are moderate, 8–9 are severe, and 10+ are extremely severe; for stress, scores from 10 to 12 are moderate, 13–16 are severe, and 17+ are extremely severe.

The BDASS-21 has demonstrated excellent psychometric properties, with Cronbach’s alphas of 0.99 for depression, 0.96 for anxiety, and 0.96 for stress [39], indicating high internal consistency. Given its strong psychometric properties, cultural relevance, and successful application in similar contexts, the BDASS-21 was chosen as the most appropriate tool to assess mental health outcomes among persons with disabilities during the COVID-19 pandemic. In the current study the scale showed a good consistency (Cronbach alpha 0.72, 0.82, 0.85 respectively).

### Statistical analysis

Stata 13 was used to analyze the data. Descriptive and inferential statistical analyses were performed to extract frequencies, percentages, and means. Chi-squares tests, independent samples *t*-tests, and one-way ANOVAs were conducted. The Chi-square test was used to analyze categorical data, including independent variables (e.g., age, gender) and dependent variables (i.e., depression, anxiety, and stress) to determine if significant associations. Levels of depression, anxiety, and stress were assessed using the summated scores of the three individual sub-scales, with higher scores indicating higher levels of depression, anxiety, and stress. Prevalence rates were calculated by dividing the number of participants with mental health conditions and the total number of participants.

Independent samples *t*-tests and one-way ANOVAs were used to compare two or more groups or conditions to determine if the groups had statistically significant mean differences. Finally, binary logistic regression was used to understand how the independent variables (e.g., age, gender) affected the likelihood of outcomes (i.e., depression, anxiety, and stress). The Shapiro-Wilk statistic was not significant, demonstrating the assumptions of normality were not violated. Levene’s test was also non-significant; thus, an equal variance could be assumed across groups.

## Results

### Demographic description of the participants

The analysis of sociodemographic variables suggests that 539 (56.7%) individuals from the selected sample had physical disabilities, 111 (11.7%) had visual disabilities, 81 (8.5%) speech and language disabilities, 24 (2.5%) a hearing disability, and 195 (20.5%) had multiple disabilities. Multiple disabilities involve more than one disability, excluding intellectual disability, autism, cerebral palsy, and Down syndrome. The mean age of the participants was 38.1 years (SD=16.64). Male participants (N=513, 54.0%) outnumbered female participants (N=437, 46.0%) (Table 1).

**Table 1:** Demographic properties of participants.

Variables	N	%
Location		
Dhaka	337	35.47
Gazipur	389	40.95
Narayanganj	224	23.58
Gender		
Male	513	54.0
Female	437	46.0
Type of Disability		
Vision	111	11.68
Hearing	24	2.53
Multidimensional	195	20.53
Physical	539	56.74
Speech	81	8.53

### Prevalence and association of mental health issues

The results showed that 67.6% of the participants had moderate to extremely severe depression, 72.6% had moderate to extremely severe anxiety, and 49.5% had moderate to extremely severe stress (Table 2). The prevalence of depression, anxiety, and stress varied significantly by location. In the rural area of Gazipur, the prevalence was exceptionally high: 86.16% for depression, 91.07% for anxiety, and 97.77% for stress. In comparison, urban areas of Dhaka had slightly lower rates of depression (76.6%) and anxiety (86.1%), whereas Narayanganj urban areas showed much lower levels of depression (54.4%) and anxiety (50.5%). Interestingly, stress prevalence was slightly higher in Narayanganj (36.7%) than in Dhaka (32.1%).

**Table 2:** Distribution of variables and relationship with depression, anxiety, and stress among people with disabilities in rural and urban areas (N=950).

Variables	Total 950	Depression (N = 642; 67.6%)				Anxiety (N = 690; 72.6%)				Stress (N = 470; 49.5%)			
	*N* (%)	Yes (%)	*χ2* value	*df*	*p* value	Yes (%)	*χ2* value	*df*	*p* value	Yes (%)	*χ2* value	*df*	*p* value
Age													
18 to 35 Years	498 (52.4)	347 (69.9)	2.893	3	0.408	355 (71.29)	4.29	3	0.232	230 (46.2)	8.19	3	0.042
36 to 50 Years	230 (24.2)	154 (67.0)				178 (77.39)				131 (57.0)			
51 to 65 Years	167 (17.6)	108 (64.7)				121 (72.46)				85 (50.9)			
>65 Years	56 (5.9)	34 (60.7)				37 (66.07)				24 (43.6)			
Gender													
Female	437 (46.0)	297 (68.0)	0.054	1	0.815	325 (74.4)	1.23	1	0.267	215 (49.2)	0.024	1	0.876
Male	513 (54.0)	345 (67.3)				365 (71.2)				255 (49.7)			
Location													
Rural	305 (32.1)	214 (70.2)	1.37	1	0.242	226 (74.1)	0.486	1	0.486	246 (80.6)	174.74	1	0.00
Urban	645 (67.9)	428 (66.4)				464 (71.9)				224 (34.7)			
Type of Disability													
Visual	111 (11.7)	76 (68.5)	3.72	4	0.445	79 (71.2)	1.179	4	0.881	61 (55.0)	8.76	4	0.007
Hearing	24 (2.5)	14 (58.3)				18 (75.0)				15 (62.5)			
Multidimensional	195 (20.5)	127 (65.3)				146 (74.9)				102 (52.3)			
Physical	539 (56.7)	364 (67.5)				391 (72.5)				262 (48.6)			
Speech and language	81 (8.5)	61 (75.3)				56 (69.1)				30 (37.0)			
Depression													
Yes	642 (67.6)	–	–	–	–	458 (71.3)	1.6629	1	0.197	470 (49.5)	1.0465	1	0.306
No	308 (32.4)	–	–	–	–	232 (75.3)				145 (47.1)			
Anxiety													
Yes	690 (72.6)	458 (66.4)	1.6629	1	0.197	–	–	–	–	402 (58.3)	77.88	1	0.00
No	260 (27.4)	184 (70.8)				–	–	–	–	68 (26.2)			
Stress													
Yes	470 (49.5)	325 (69.2)	1.0465	1	0.306	402 (85.5)	77.88	1	0.00	–	–	–	–
No	480 (50.3)	317 (66.0)				288 (60.0)				–	–	–	–

Age (χ2 = 8.19, df = 3, p < 0.042) and geographical location (χ2 = 174.74, df = 1, p < 0.001) were significantly associated with stress. Younger participants, particularly in rural areas, experienced significantly higher levels of stress, suggesting that this group might be more vulnerable to stressors. Additionally, participants with greater levels of anxiety were significantly more likely to experience higher levels of stress (χ2 = 77.88, df = 1, p < 0.001). Disability type was also significantly associated with stress (χ2 = 8.76, df = 4, p < 0.007), with individuals having hearing disabilities experiencing higher stress levels than those with other disabilities. Depression and anxiety, however, were not significantly associated with age, gender, geographical location, or type of disability (see Table 2).

### Independent samples t-tests for gender and geographical location about mental health

Results of independent samples *t*-tests demonstrated that there were no significant differences in depression scores for male (M=8.66, SD=4.19) and female participants (M=9.08, SD=4.22; *t*(949)=-1.535, *p*=.125) or stress scores (Males: M=9.94, SD=4.45; Females: M=9.96, SD=4.81; *t*(949)=-1.533, *p* =.69). However, males (M=7.66, SD=3.96) reported significantly lower levels of anxiety compared to female participants (M=8.18, SD=3.83; *t*(949)=-1.533, *p* <.05; See Table 2). Results of *t*-*t*est also indicated that there was a statistically significant difference in the mean scores of depression, anxiety, and stress between urban and rural participants (see Tables 3 and 4).

**Table 3:** Independent samples t-tests for gender and geographical location in relation to Depression, Anxiety, and Stress.

Gender difference									
Depression	Male (N=513)		Female (N=437)		*F*	*t*	df	*p*	Cohen’d
	M	SD	M	SD					
	8.66	4.19	9.08	4.22	.001	−1.535	949	.125	.10
Anxiety	Male (N=513)		Female (N=437)						
	M	SD	M	SD					
	7.66	3.96	8.18	3.83	.502	−1.533	949	.04	.13
Stress	Male (N=513)		Female (N=437)						
	M	SD	M	SD					
	9.94	4.45	9.96	4.81	5.073	−1.533	949	.69	.004
**Geographical Location**									
Depression	Urban (N=642)		Rural (N=309)		*F*	*t*	df	*p*	Cohen’s d
	M	SD	M	SD					
	9.64	4.37	7.24	3.32	39.97	8.536	949	.000	.62
Anxiety	Urban (N=642)		Rural (N=309)						
	M	SD	M	SD					
	8.72	3.86	6.20	3.44	11.22	9.767	949	.000	.69
Stress	Urban (N=642)		Rural (N=309)						
	M	SD	M	SD					
	10.59	4.91	8.46	3.55	72.86	6.831	949	.000	.50

**Table 4:** Binary logistic regression coefficients of sociodemographic variables on depression, anxiety, and stress.

Depression								
**Variables**	***B***	***SE B***	**Wald** $\chi^{2}$	***df***	***p***	***OR***	**95% CI OR**	
							**LL**	**UL**
Age	−0.002	0.004	0.251	1	0.616	0.998	0.990	1.006
Gender (ref. Male)	−0.131	0.142	0.860	1	0.354	0.877	0.664	1.158
Disability (ref. Physical)								
Speech and Language	−0.211	0.187	1.272	1	0.259	0.810	0.562	1.168
Visual	−0.788	0.276	8.156	1	0.004	0.455	0.265	0.781
Hearing	−0.064	0.266	0.057	1	0.811	0.938	0.557	1.582
Multiple	0.598	0.571	1.096	1	0.295	1.818	0.594	5.569
Location (ref. Urban)	0.761	0.147	26.836	1	0.000	2.141	1.605	2.856
**Anxiety**								
**Variables**	***B***	***SE B***	**Wald** $\chi^{2}$	***df***	***p***	***OR***	**95% CI OR**	
							**LL**	**UL**
Age	0.002	0.004	0.221	1	0.638	1.002	0.993	1.011
Gender (ref. Male)	−0.166	0.147	1.278	1	0.258	0.847	0.636	1.129
Disability (ref. Physical)								
Speech and Language	−0.093	0.19	0.24	1	0.624	0.911	0.627	1.323
Visual	−0.258	0.291	0.787	1	0.375	0.772	0.437	1.367
Hearing	−0.161	0.266	0.366	1	0.545	0.851	0.505	1.434
Multiple	0.545	0.572	0.908	1	0.341	1.724	0.562	5.287
Location (ref. Urban)	1.143	0.152	56.663	1	0.000	3.136	2.329	4.223
**Stress**								
**Variables**	***B***	***SE B***	**Wald** $\chi^{2}$	***df***	***p***	***OR***	**95% CI OR**	
							**LL**	**UL**
Age	0.005	0.004	1.938	1	0.164	1.005	0.998	1.013
Gender (ref. Male)	0.017	0.130	0.017	1	0.896	1.017	0.788	1.313
Disability (ref. Physical)								
Speech and Language	−0.004	0.167	0.001	1	0.979	0.996	0.717	1.382
Visual	−0.479	0.271	3.127	1	0.077	0.619	0.364	1.053
Hearing	0.214	0.238	0.805	1	0.370	1.238	0.776	1.976
Multiple	1.997	0.634	9.935	1	0.002	7.368	2.128	25.510
Location (ref. Urban)	0.729	0.142	26.397	1	0.000	2.074	1.570	2.739

Note. OR = odds ratio. CI = confidence interval

Based on the Tukey HSD test results, persons with multiple disabilities (M=10.07, SD=4.59) and those with a hearing disability (M=12.46, SD=5.70) reported higher levels of depression compared to those with a physical disability (M=8.59, SD=3.89, *p*<.05), visual disability (M=8.36, SD=3.70, *p*<.05), and those a speech and language impairment (M=7.35, SD=4.33, *p*<.05).

Persons with a hearing disability reported greater anxiety levels (M=10.25, SD=3.84) compared to those with a physical disability (M=7.89, SD=3.87; *p*=.03) and those with a speech and language disability (M=6.80, SD=3.30; *p*<.05). No other groups were significantly different.

Persons with a hearing disability reported greater levels of stress (M=14.21, SD=4.43) compared to those with a physical disability (Mean=9.89, SD=4.66, *p*<.05), speech and language disability (M=8.49, SD=4.18; *p*<.05), visual disability (M=10.13, SD=4.61; *p*<.05), and those with multiple disabilities (M=9.82, SD=4.42; *p*<.05).

## Discussion

The COVID-19 pandemic has significantly impacted global mental health, with individuals facing various stressors such as isolation, uncertainty, and disrupted routines as well as severe mental health disorders [40–42]. Persons with disabilities, who often experience additional challenges related to healthcare access, mobility, and social participation, have been disproportionately affected by the pandemic [43]. In Bangladesh, where healthcare infrastructure and support systems for people with disabilities are limited, understanding the specific mental health implications for this vulnerable group during the pandemic is critical for addressing their needs and improving future responses [44]. Few studies to date have explored the mental health problems of persons with disabilities in the rural and urban areas of Bangladesh, therefore the present study aimed to address this gap. The study used the widely recognized DASS-21 to assess common mental health markers (i.e., depression, anxiety, and stress). While the DASS-21 has been validated with several populations (e.g., autism spectrum disorder [45], borderline intellectual disability [46], little evidence exists on the prevalence of depression, anxiety, and stress among persons with disabilities using the DASS-21, especially during the COVID-19 pandemic.

The findings of the present study suggest that persons with disabilities experienced mental health problems such as depression, anxiety, and stress during the pandemic. Comparable data indicating the level of depression, anxiety, and stress among persons with disabilities elsewhere in the world, including Bangladesh, are scarce. A recent study reported symptoms of depression, anxiety, and stress among people with disabilities in rural areas [44]. The findings of the present study converge with the findings from Faruk et al. (2024), namely factors such as lack of access to mental health care, compromised physical and/or mental health status, and socioeconomic barriers may impact the high levels of depression, anxiety, and stress reported by this population.

The present study found no statistical association of gender with depression or stress, consistent with an earlier study [47]. Women did report higher levels of anxiety compared to men. Prior studies have found that women experience a greater burden of disability due to the higher prevalence of depression and anxiety disorders in this population [16,47]. Sultana and Gulshan (2014) [48] found an association between gender and mental health problems, with females with disabilities in Bangladesh experiencing more mental health issues. However, gender differences in the association between mental health problems and disability is believed to be inconclusive due to the higher reliance on clinical studies, which include a small number of overall participants and a greater inclusion of female participants [47].

The results also indicated that certain types of disabilities, such as hearing disability and multiple disabilities, were associated with higher levels of depression and stress [49]. Disabilities may limit a person's ability to engage in activities important for mental well-being, such as socializing, exercising, and pursuing hobbies. Additionally, the experience of living with a disability can result in daily challenges and limitations that can lead to increased stress, exacerbated by factors like restricted movement during the pandemic and disability-related stigma and discrimination.

Stress, but not depression or anxiety, differed significantly across ages, irrespective of disability status experience stress during the pandemic [16,50–52]. Stress was also found to be associated with geographical location and type of disability, with urban participants experiencing more mental health problems than their rural counterparts. This disparity may be attributed to factors such as widespread stigma and discrimination, as well as a lack of mental health care facilities in both rural and urban areas of Bangladesh. Further exploration is needed to understand the factors responsible for the higher prevalence of mental health problems among persons with disabilities in urban areas.

Although research on the associations between types of disability and mental health outcomes is limited, available studies indicate that certain types of disabilities may lead to increased symptoms of depression and anxiety [53–57]. However, persons with disabilities may experience higher levels of stress due to societal and environmental factors such as discrimination, lack of accessibility, and limited opportunities for employment and social participation. The COVID-19 pandemic may have intensified these existing challenges, potentially leading to a significant increase in mental health issues among this population. Further research is needed to address this gap and document mental health markers among persons with varying types of disabilities during the pandemic.

## Implications of the study

This study is the first to examine the mental health status of persons with disabilities during the COVID-19 pandemic in Bangladesh, and its findings are consistent with global research that highlights the increased mental health risks faced by individuals with disabilities during such crises. The prevalence of mental health issues among this population in both urban and rural Bangladesh calls for urgent policy attention. Globally, it is well-documented that persons with disabilities experience heightened levels of depression, anxiety, and stress during emergencies, exacerbated by factors like isolation, lack of healthcare access, and disruption of daily routines. In light of these findings, there are clear implications for disability-inclusive policy development in Bangladesh. The country's existing disability rights laws, such as the Persons with Disabilities Rights and Protection Act, do not include provisions for addressing the mental health needs of this group during emergencies. This gap in the policy framework underscores the need for comprehensive revisions to ensure that future policies incorporate mental health support for persons with disabilities in times of crisis. A key recommendation is the integration of emergency preparedness plans that specifically address the mental health and well-being of this vulnerable group, ensuring their access to appropriate mental health services, even during health emergencies like pandemics.

## Limitations

The present study has several limitations. Due to its cross-sectional design, causal relationships between mental health problems and sociodemographic factors cannot be inferred. Additionally, the use of purposive sampling limits the study’s representativeness. Since participants were selected based on specific characteristics, the findings may not fully reflect the broader population of persons with disabilities. Future research should employ random or stratified sampling to improve representativeness.

The study also did not include individuals with all types of disabilities, which further limits the generalizability of the findings. Self-reported measures may introduce response biases, such as social desirability and researchers’ bias, which could affect the data’s accuracy. A disproportionate representation of urban versus rural participants (with more urban participants) may limit the external validity of the results. A nationwide study with proportionate sampling of urban and rural areas is recommended for future research.

While the DASS-21 is widely used [32–37], it has not been validated for children and adolescents under 17. Despite this, the DASS-21 has been used in young adults with autism and low vision [45,58]. The study found three factors consistent with the original scale, supporting its robustness in diverse populations. Future studies should validate the DASS-21 for persons with disabilities or use other validated scales.

Lastly, the lack of pre- and post-comparisons limits the ability to assess changes in mental health over time during the COVID-19 pandemic. Without baseline and follow-up data, the impact of the pandemic on individuals with disabilities cannot be fully determined. Although the study offers valuable insights, its lack of longitudinal data restricts understanding of the temporal relationship between disability status and mental health outcomes.

A longitudinal study design would be beneficial to explore the long-term mental health impacts on individuals with disabilities. Future research could investigate the relationship between mental health problems and various factors, such as disability type, as well as the social, attitudinal, personal, situational, and structural variables that may influence these outcomes. Such studies would help develop more comprehensive, targeted interventions and policies that address the specific needs of individuals with disabilities.

## Conclusion

The current study reveals a high prevalence of depression, anxiety, and stress among individuals with disabilities in both rural and urban areas of Bangladesh during the pandemic. Specifically, the study found that 67.6% of participants experienced moderate to extremely severe depression, 72.6% had moderate to extremely severe anxiety, and 49.5% reported moderate to extremely severe stress. These findings underscore the critical need for targeted mental health interventions for persons with disabilities, especially considering the geographical disparities observed. Rural areas, in particular, displayed higher rates of mental health issues, highlighting gaps in mental health service accessibility. The study’s findings emphasize the importance of developing specialized mental health services tailored to the unique needs of persons with disabilities in Bangladesh, with a focus on addressing both the individual and environmental factors contributing to their mental health struggles. These results have significant policy implications, urging the government to revise existing frameworks and policies to better accommodate the mental health needs of persons with disabilities, especially during health crises like the COVID-19 pandemic.

## Supporting information

S1 File Sample data. (SAV)
